# Matrix metalloproteinases as diagnostic and prognostic biomarkers in skin cutaneous melanoma and squamous cell carcinoma

**DOI:** 10.1038/s41598-025-06608-3

**Published:** 2025-07-02

**Authors:** Jingya Hei, Xiaohui Bai, Jing Wang

**Affiliations:** 1 Department of Pathology, The General Hospital of Ningxia Medical University, YinChuan, 750004 NingXia China; 2 Emergency Department, Third People’s Hospital of Ningxia Autonomous Region, Yinchuan, 750027 NingXia China

**Keywords:** Matrix metalloproteinases, Skin cutaneous melanoma, Squamous cell carcinoma, ECM remodeling, Diagnostic biomarkers, Cancer, Squamous cell carcinoma

## Abstract

Skin cutaneous melanoma (SKCM) and squamous cell carcinoma (SCC) are two prevalent forms of skin cancer, both characterized by extensive remodeling of the extracellular matrix (ECM), which facilitates tumor progression and metastasis. To investigate ECM-related gene expression alterations in SCC and assess their diagnostic and prognostic relevance in SKCM by integrating transcriptomic data with clinical outcomes. RNA-sequencing data from NCBI Gene Expression Omnibus (GEO) were analyzed to identify differentially expressed genes in SCC. Key findings were validated in The Cancer Genome Atlas (TCGA) SKCM dataset. Functional enrichment and protein–protein interaction (PPI) analyses were conducted to identify ECM-related pathways and hub genes. Matrix metalloproteinases MMP7, MMP11, and MMP14 were significantly upregulated in SCC and showed elevated expression in primary SKCM tumors, confirmed by RT-qPCR analysis. Functional enrichment revealed dysregulation of ECM–receptor interaction and IL-17 signaling pathways. PPI analysis identified MMP14 as central hub interacting with TIMPs, CD44, and FURIN. Kaplan–Meier survival analysis showed that elevated MMP11 and MMP14 expression correlated with worse overall survival. ROC curve analysis confirmed their strong diagnostic value, with MMP14 achieving an AUC of 0.955. MMP7, MMP11, and MMP14 play key roles in skin cancer progression. They show strong potential as diagnostic and prognostic biomarkers and may serve as therapeutic targets in SCC and SKCM.

## Introduction

Skin cutaneous melanoma and squamous cell carcinoma are significant skin malignancies with distinct characteristics and treatment challenges. SKCM, originating from melanocytes, is highly aggressive and prone to metastasis, making early detection and treatment crucial. Treatments may include surgery, immunotherapy, targeted therapy, and chemotherapy, though their effectiveness can be limited by disease stage, tumor location and patient response^1–3^.

In contrast, SCC originates in the squamous cells of the epidermis. SCC typically arises from chronic ultraviolet (UV) exposure, leading to DNA damage and disruption of genes regulating proliferation and differentiation. Although less likely to metastasize than melanoma, SCC can still exhibit aggressive behavior if left untreated. SCC is primarily treated through surgical removal, with additional options such as cryotherapy, topical chemotherapy, and radiation therapy depending on the location and severity of the tumor^4,5^.

According to the GLOBOCAN 2022 report, skin cancers, including SKCM and non-melanoma types like SCC, represent a significant global health concern, with nearly half a million new cancer cases annually and approximately 58,667 cancer deaths worldwide^6^. SKCM and SCC, despite differing in their genetic alterations, both involve significant changes to the ECM that facilitate tumor progression. It is a complex network of proteins and other molecules that provide structural support to tissues. In skin cancer, degradation of the ECM is a critical process that facilitates tumor progression and metastasis^7–9^. This degradation is primarily mediated by enzymes such as MMPs, which break down ECM components like collagen and elastin.

Dysregulated ECM significantly influences progression and response to treatment. Enzymes such as MMP-1, MMP-2, MMP-9, MMP-13 and MMP-14, play critical roles in ECM remodeling. MMP-14, in particular, is known to activate MMP-2, enhancing the invasive potential of melanoma cells. High expressions of MMP-14 and MMP-2 correlate with poor prognosis and are prevalent in aggressive melanoma subtypes^8–10^.

Matrix metalloproteinases (MMPs), a family of zinc-dependent endopeptidases, play a pivotal role in ECM degradation. In addition to degrading structural components such as collagen and elastin, MMPs are now recognized as key modulators of the tumor microenvironment (TME). They regulate immune infiltration, cytokine activation, and stromal remodeling, thereby influencing cancer cell behavior, angiogenesis, and response to therapy^10–13^. Recent pan-cancer analyses have further emphasized that MMPs interact with immune checkpoint pathways and cancer-associated fibroblasts (CAFs), reinforcing their role as central players in TME remodeling^14^.

Both melanoma and SCC demonstrate how ECM remodeling can influence resistance to therapeutic interventions. In melanoma, altered ECM structures can impede drug delivery and immune cell infiltration, reducing the effectiveness of targeted therapies and immune checkpoint inhibitors^14–16^. Similarly, in SCC, ECM remodeling by CAFs and changes in ECM composition, such as increased fibronectin and tenascin C, can alter the tumor microenvironment, contributing to therapeutic resistance. These changes impact the effectiveness of treatments by affecting drug penetration and promoting a proinflammatory milieu that supports tumor progression. The overlapping mechanisms of ECM remodeling in SKCM and SCC underscore the therapeutic value of targeting the ECM to overcome resistance and improve therapeutic outcomes^17–20^.

Given the scarcity of studies comparing SKCM and SCC and recognizing the crucial role of ECM remodeling and dysfunction, this study aimed to utilize RNA sequencing to identify biomarkers associated with ECM degradation in these skin cancers. By analyzing gene expression profiles and pinpointing dysregulated genes and pathways linked to ECM remodeling, the study seeks to enhance the understanding of ECM dysregulation in SKCM and SCC.

## Material and methods

### Data collection and differential expression analysis

Transcriptomic data were retrieved from the NCBI Gene Expression Omnibus (GEO) under the accession number GSE191334, which includes RNA-sequencing data of human SCC and normal skin tissue. The dataset comprises a total of 16 samples, including 8 SCC samples and 8 normal skin samples, derived from skin biopsies. The sequencing was conducted using the Illumina HiSeq 4000 platform, employing paired-end reads to ensure high sequencing depth and transcriptome coverage.

The patient cohort includes individuals diagnosed with cutaneous SCC, and the control samples represent non-cancerous skin tissues from age-matched healthy individuals. Although the original dataset does not include detailed demographic or clinical metadata (e.g., age, sex, tumor stage), all samples were processed under standardized experimental protocols.

Raw read count matrices were downloaded and processed using the DESeq2 package (v1.36.0) in R version 4.2.2, which models count data using a negative binomial distribution. Data normalization was carried out using the median of ratios method. Genes with an absolute log2 fold change (|log2FC|) > 1 and adjusted *p*-value < 0.05 were considered differentially expressed. Results were visualized using volcano plots, and significant gene sets were subjected to downstream pathway enrichment analysis.

To validate findings in melanoma, we utilized the TCGA-SKCM dataset from the UCSC Xena platform. This dataset includes primary and metastatic tumor samples, with limited normal tissue controls (n = 2). Due to this limitation, differential expression analysis focused on comparing primary versus metastatic tumors. Normalized gene expression values (log2-transformed) were extracted from the TCGA Pan-Cancer (PANCAN) cohort for downstream validation.

### Pathway enrichment analysis

Functional enrichment analysis was performed using the enrichR package in R (version 4.2.2), focusing on Kyoto Encyclopedia of Genes and Genomes (KEGG) pathways. Upregulated and downregulated genes were analyzed separately, and significant pathways were visualized using dot plots sorted by adjusted *p*-values. Pathways related to extracellular matrix (ECM) remodeling were prioritized for downstream analysis^21^.

### Protein–protein network analysis

To investigate potential functional relationships among ECM-associated genes, PPI analysis was performed using the STRING database. Genes enriched in ECM-related pathways were input into the STRING tool under default settings. Network clustering was used to identify key functional modules. The largest cluster with direct involvement in ECM degradation was selected for further validation^22,23^.

### Validation using TCGA-SKCM dataset

To assess the relevance of selected ECM-related genes in melanoma, expression levels were analyzed in the TCGA Skin Cutaneous Melanoma (SKCM) dataset via the UCSC Xena platform. Due to limited normal skin samples (n = 2), comparisons were made between primary and metastatic tumor samples. Genes showing reduced expression in metastasis were excluded, while those with elevated expression in primary tumors were retained^24^.

### Survival analysis

The prognostic significance of MMP7, MMP11, and MMP14 was evaluated using the GEPIA2 platform, which integrates data from TCGA and GTEx. Kaplan–Meier survival curves were generated for overall survival (OS), stratifying patients into high- and low-expression groups based on median gene expression. Statistical significance was determined using the log-rank test.

### ROC curve analysis

Diagnostic performance of MMP7, MMP11, and MMP14 in SKCM was assessed using ROC curve analysis in GraphPad Prism 7.0 and the pROC package in R. Area under the curve (AUC), sensitivity, and specificity were calculated. An AUC > 0.7 was considered indicative of acceptable diagnostic accuracy.

### Promoter methylation analysis

Promoter methylation levels of MMP7, MMP11, and MMP14 were analyzed using the MEXPRESS platform, with validation using UCSC Xena. Methylation status was correlated with mRNA expression levels to explore potential epigenetic regulatory mechanisms.

### Immune and functional correlation analysis

Correlations between MMP expression and immune checkpoint genes, as well as immune cell infiltration levels, were assessed using the TISIDB and GSCA platforms. Additionally, CancerSEA was used to investigate single-cell functional states such as epithelial–mesenchymal transition (EMT), angiogenesis, invasion, and metastasis.

### Experimental validation in SKCM cell lines

Functional validation was conducted using A375 melanoma cell lines (obtained from the Cell Bank of the Chinese Academy of Sciences, Shanghai, China). Cells were transfected with small interfering RNA (siRNA) targeting MMP7 using Lipofectamine RNAiMAX (Thermo Fisher Scientific, USA). Knockdown efficiency was confirmed by RT-qPCR and Western blotting.

### Cell proliferation assay

Cell viability was assessed using the MTS assay at 24, 48, and 72 h post-transfection.

### Colony formation assay

Colony-forming ability was measured after 10 days of culture, followed by crystal violet staining.

### Wound healing assay

Cell migration was evaluated using a scratch wound healing assay. Images were captured at 0 and 24 h.

### Quantitative real-time PCR (qRT-PCR) analysis

Total RNA was extracted using TRIzol reagent (Invitrogen, USA), and RNA concentration and purity were measured using a NanoDrop spectrophotometer (Thermo Fisher Scientific, USA). RNA integrity was verified via the Agilent 2100 Bioanalyzer. First-strand cDNA synthesis was performed using the RevertAid First Strand cDNA Synthesis Kit.

The qRT-PCR was carried out using the ABI 7900HT Fast Real-Time PCR System with SYBR Green PCR Master Mix. GAPDH served as an internal control. Primer sequences are listed below^23^ (Table 1).

**Table 1:** Primer Sequences Used for RT-qPCR.

Gene	Forward Primer (5′ → 3′)	Reverse Primer (5′ → 3′)
GAPDH	ACCCACTCCTCCACCTTTGAC	CTGTTGCTGTAGCCAAATTCG
MMP7	TCGGAGGAGATGCTCACTTCGA	GGATCAGAGGAATGTCCCATACC
MMP11	GAGAAGACGGACCTCACCTACA	CTCAGTAAAGGTGAGTGGCGTC
MMP14	CCTTGGACTGTCAGGAATGAGG	TTCTCCGTGTCCATCCACTGGT

Amplicons ranged from 100 to 150 base pairs. Primer specificity was verified via NCBI Primer-BLAST. All reactions were run in triplicate.

### Statistical analysis

All statistical analyses were performed using R version 4.2.2 and GraphPad Prism 7.0. Group comparisons were made using Student’s t-test. Survival differences were assessed using the log-rank test. ROC curve analysis employed the pROC package and GraphPad software. Spearman’s correlation was used to analyze gene co-expression relationships. A *p*-value < 0.05 was considered statistically significant.

## Results

### Differential gene expression in SCC

**Fig. 1:**
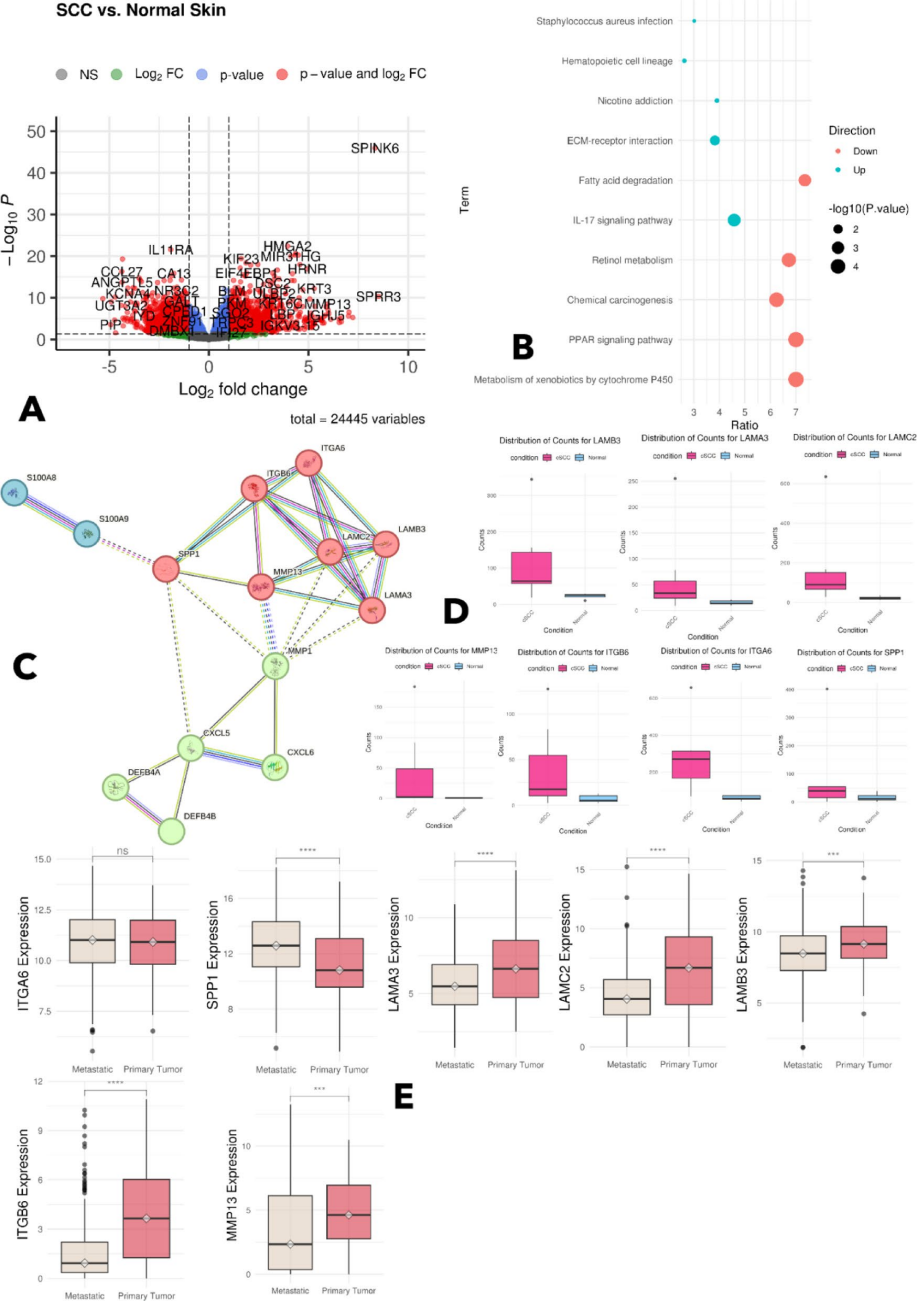
(**A**) Volcano plot depicting the differentially expressed genes between SCC and normal skin samples. (**B**) Functional enrichment analysis of differentially expressed genes revealed upregulation of ECM–receptor interaction and IL-17 signaling pathways^25,26^. (**C**) PPI are clustered based on functional closeness to each other, the red cluster being the biggest one and chosen for analysis. (**D**) Differential normalized count values between SCC and normal skin samples indicate significant upregulation of the genes in SCC. E. Validation of the differentially expressed genes based on TCGA data indicates that most of the shortlisted genes are highly upregulated in primary tumors of the skin and become downregulated in the metastatic stage^25,26^.

We identified differentially expressed genes between squamous cell carcinoma (SCC) and normal skin tissue using DESeq2. A total of 1448 genes were significantly upregulated, and 1,700 were downregulated (*p* < 0.05) (Fig. 1A).

### ECM-related gene alterations and pathway enrichment

Functional enrichment analysis of dysregulated genes revealed significant involvement in ECM–receptor interaction and IL-17 signaling pathways. Several ECM-related genes, including LAMB3, LAMA3, SPP1, and ITGB6, were notably upregulated (Fig. 1B).

### Selection and validation of MMPs as key regulators

Although multiple ECM genes were dysregulated, we focused on MMP7, MMP11, and MMP14 due to their roles in both ECM remodeling and immune regulation, as well as their central positions in the PPI network (Fig. 1C–E).

### PPI network analysis

**Fig. 2:**
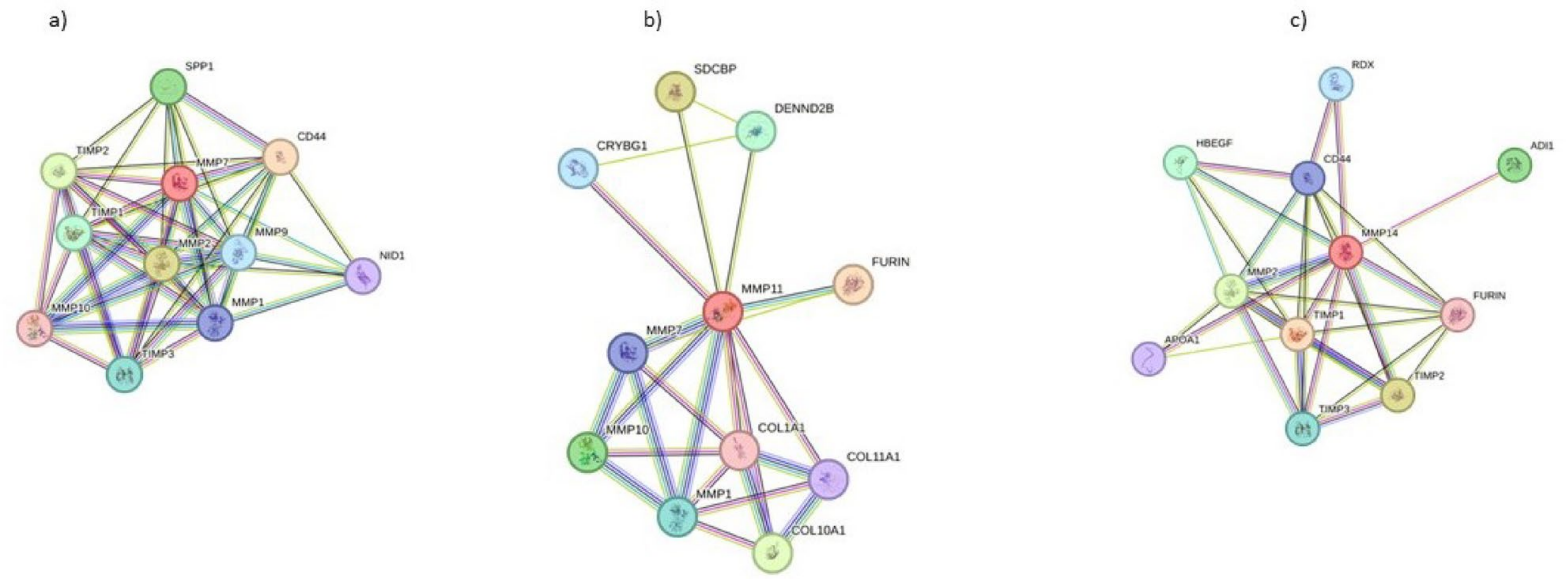
PPI networks of MMP7, MMP11, and MMP14 constructed using STRING database. (**a**) MMP7-centered network: Displays interactions with several matrix-degrading enzymes (MMP1, MMP2, MMP3, MMP9, MMP10), tissue inhibitors (TIMP1, TIMP2), and ECM receptors (CD44, SPP1). (**b**) MMP11-centered network: Highlights interactions with other MMPs (MMP2, MMP10), collagens (COL1A1, COL10A1, COL11A1), and regulatory proteins (FURIN, SDCBP), suggesting a broader role in collagen degradation and activation. (**c**) MMP14-centered network: Shows strong interactions with ECM proteins (TIMP1/2, CD44, HBEGF), and processing enzymes (FURIN), indicating a central role in ECM remodeling, cell adhesion, and tumor invasion.

STRING-based analysis showed that MMP7, MMP11, and MMP14 formed dense interaction networks with other ECM-related genes, indicating their regulatory significance in SCC and melanoma progression (Fig. 2A–C).

### Prognostic significance of MMPs in SKCM

**Fig. 3:**
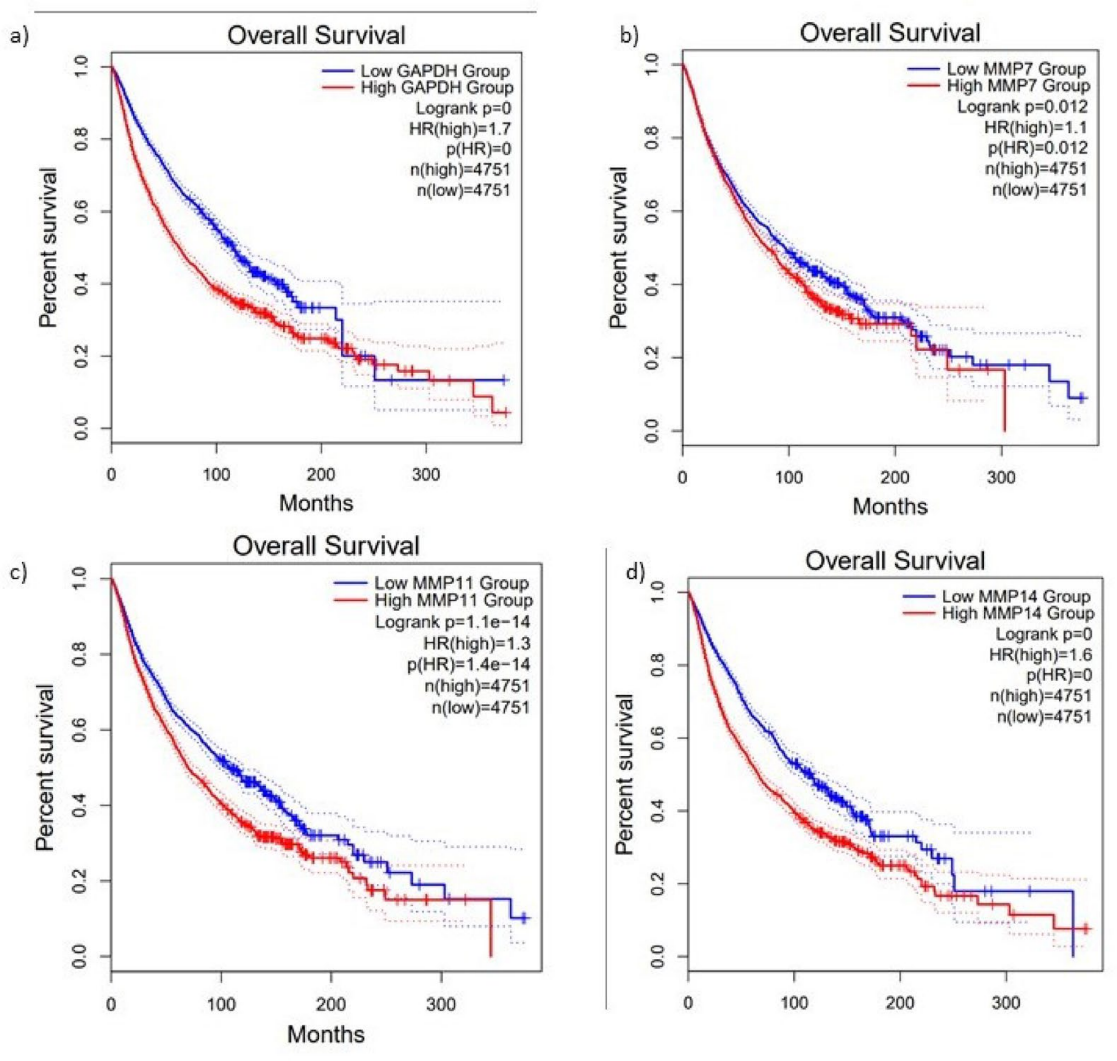
Kaplan–Meier OS analysis of GAPDH and MMPs in TCGA-SKCM. (**a**) Higher GAPDH expression was associated with significantly worse overall survival (HR = 1.7, Log-rank *p* = 0). (**b**) High MMP7 expression correlated with reduced survival (HR = 1.1, Log-rank *p* = 0.012). (**c**) Increased MMP11 expression was significantly linked to poorer survival outcomes (HR = 1.3, Log-rank p = 1.1e-14). (**d**) High MMP14 expression showed the strongest association with poor survival (HR = 1.6, Log-rank *p* = 0).

Kaplan–Meier survival analysis using GEPIA2 revealed that high expression levels of MMP7, MMP11, and MMP14 were significantly associated with worse overall survival in SKCM patients (Fig. 3A–D).

### Isoform and protein domain structure of MMPs

**Fig. 4:**
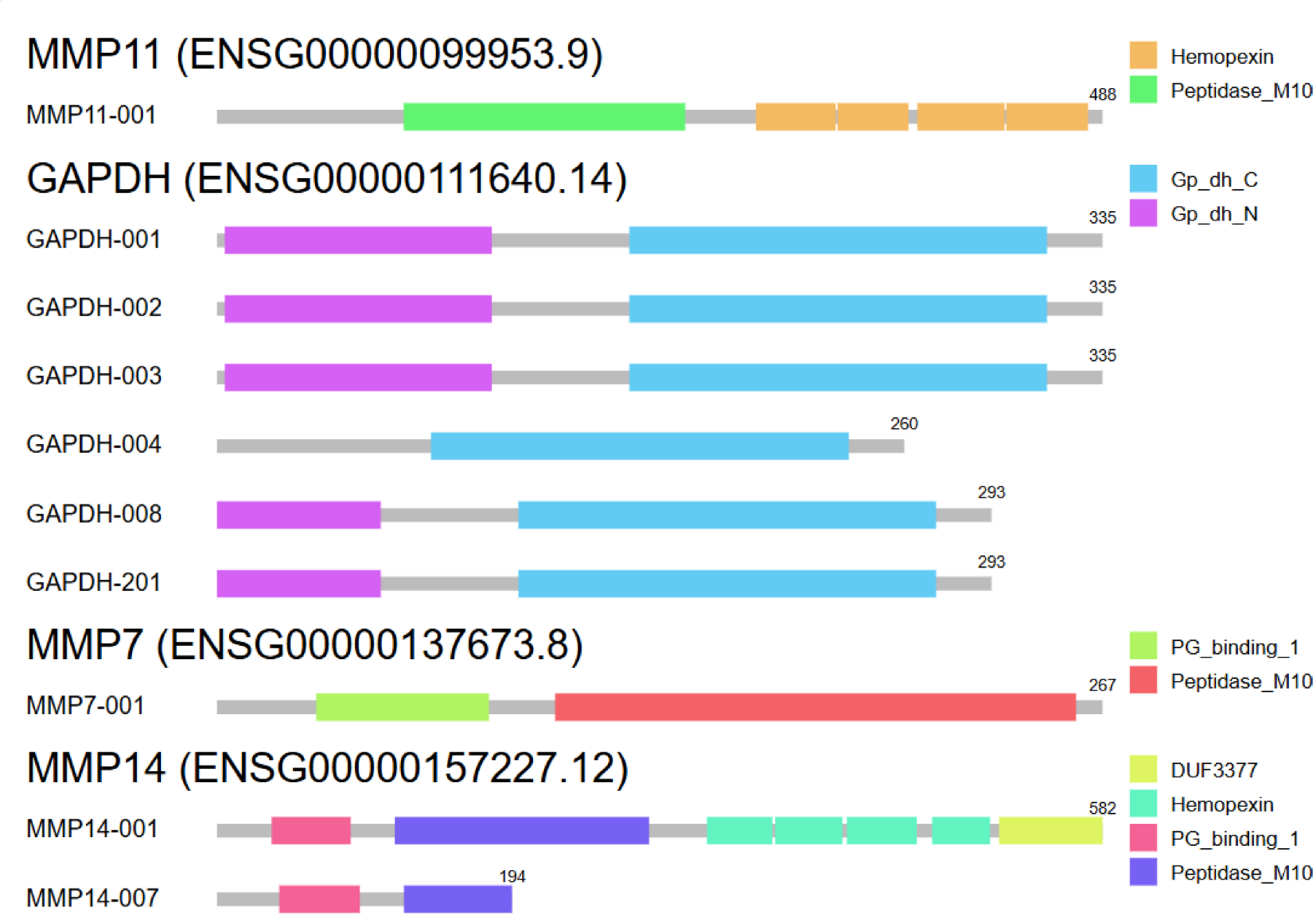
Isoform and protein domain structure analysis of MMP7, MMP11, MMP14 and GAPDH. (**a**) MMP11-001 contains Peptidase_M10 (green) and Hemopexin (orange) domains. (**b**) GAPDH isoforms (001, 002, 003, 004, 008, 201) contain Gp_dh_C (cyan) and Gp_dh_N (purple) domains. (**c**) MMP7-001 includes PG_binding_1 (green) and Peptidase_M10 (red), supporting its role in extracellular matrix remodeling. (**d**) MMP14 isoforms (001 and 007) contain multiple functional domains, including DUF3377, Hemopexin, PG_binding_1 and Peptidase_M10, highlighting its complex role in matrix degradation and cell invasion.

Isoform analysis indicated functional domains responsible for ECM degradation, such as Peptidase_M10 and Hemopexin, supporting their biological roles (Fig. 4).

### Diagnostic performance of MMPs via ROC analysis

**Fig. 5:**
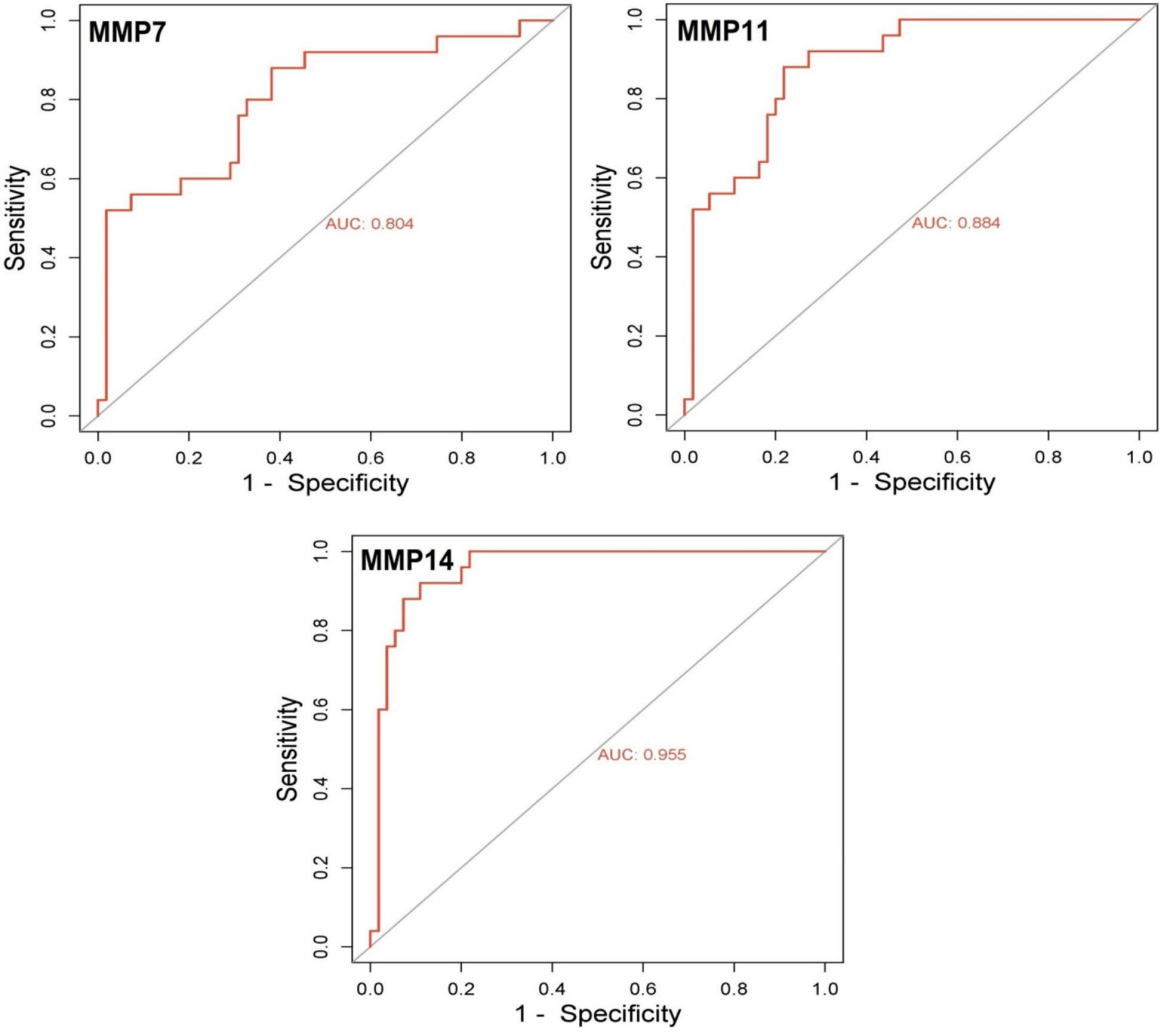
ROC curve analysis of MMP7, MMP11, and MMP14 for diagnostic performance in SKCM. (**a**) MMP7 demonstrates strong diagnostic performance with an AUC of 0.945. (**b**) MMP11 shows excellent discriminative power with an AUC of 0.993. (**c**) MMP14 shows robust separation between tumor and normal samples with an AUC of 0.955, indicating high diagnostic potential, though not perfect.

ROC curve analysis revealed excellent diagnostic performance for the three MMPs: MMP7 (AUC = 0.945), MMP11 (AUC = 0.993), and MMP14 (AUC = 0.955), supporting their value as diagnostic biomarkers in SKCM. These ROC results were derived exclusively from the TCGA dataset and lack external validation, necessitating further studies to confirm diagnostic robustness. ROC curves were generated using normalized expression data, and no external cross-validation or independent datasets were applied. Future studies should consider independent cohorts to validate these findings (Fig. 5).

### Correlation between GAPDH and MMP expression

**Fig. 6:**
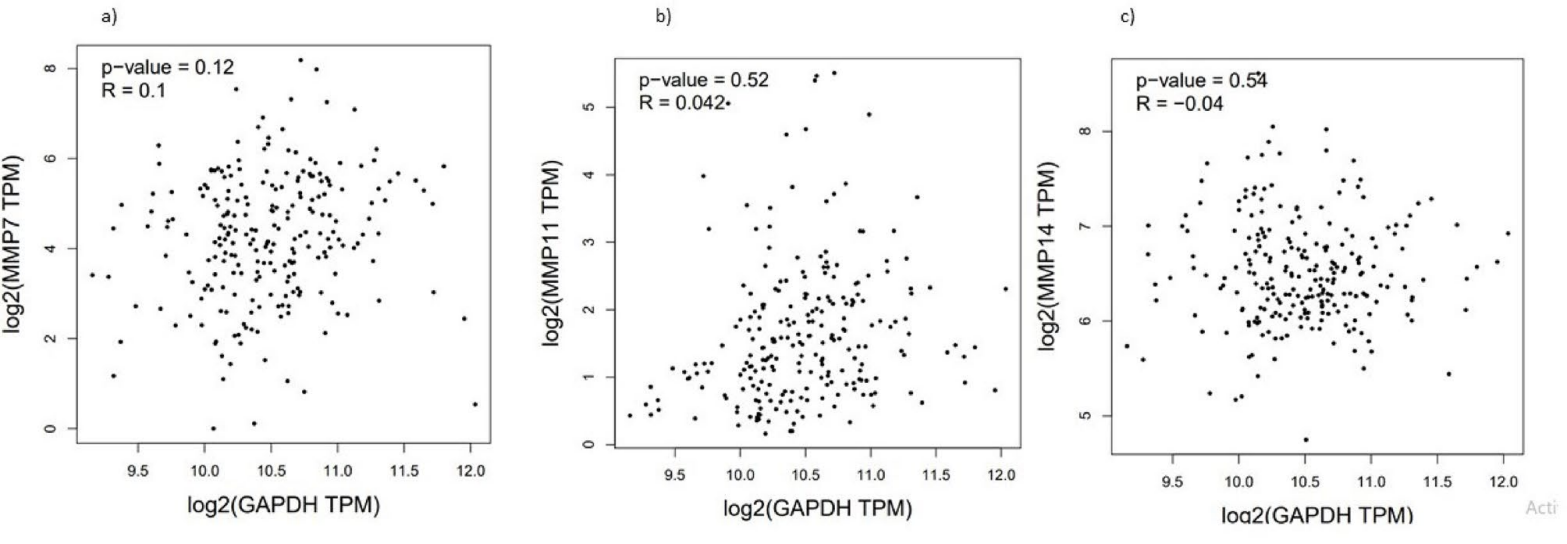
Correlation analysis of MMP expression with GAPDH in TCGA-SKCM. (**a**) MMP7 vs. GAPDH (R = 0.1, *p* = 0.12), (**b**) MMP11 vs. GAPDH (R = 0.042, *p* = 0.52), and (**c**) MMP14 vs. GAPDH (R = − 0.04, *p* = 0.54). Correlation analysis was conducted using GEPIA2.

Pearson correlation analysis revealed weak, non-significant associations between GAPDH and MMP expression levels, suggesting no direct co-regulation (Fig. 6A–C).

### MMP expression in TP53-mutant vs. wild-type SKCM

**Fig. 7:**
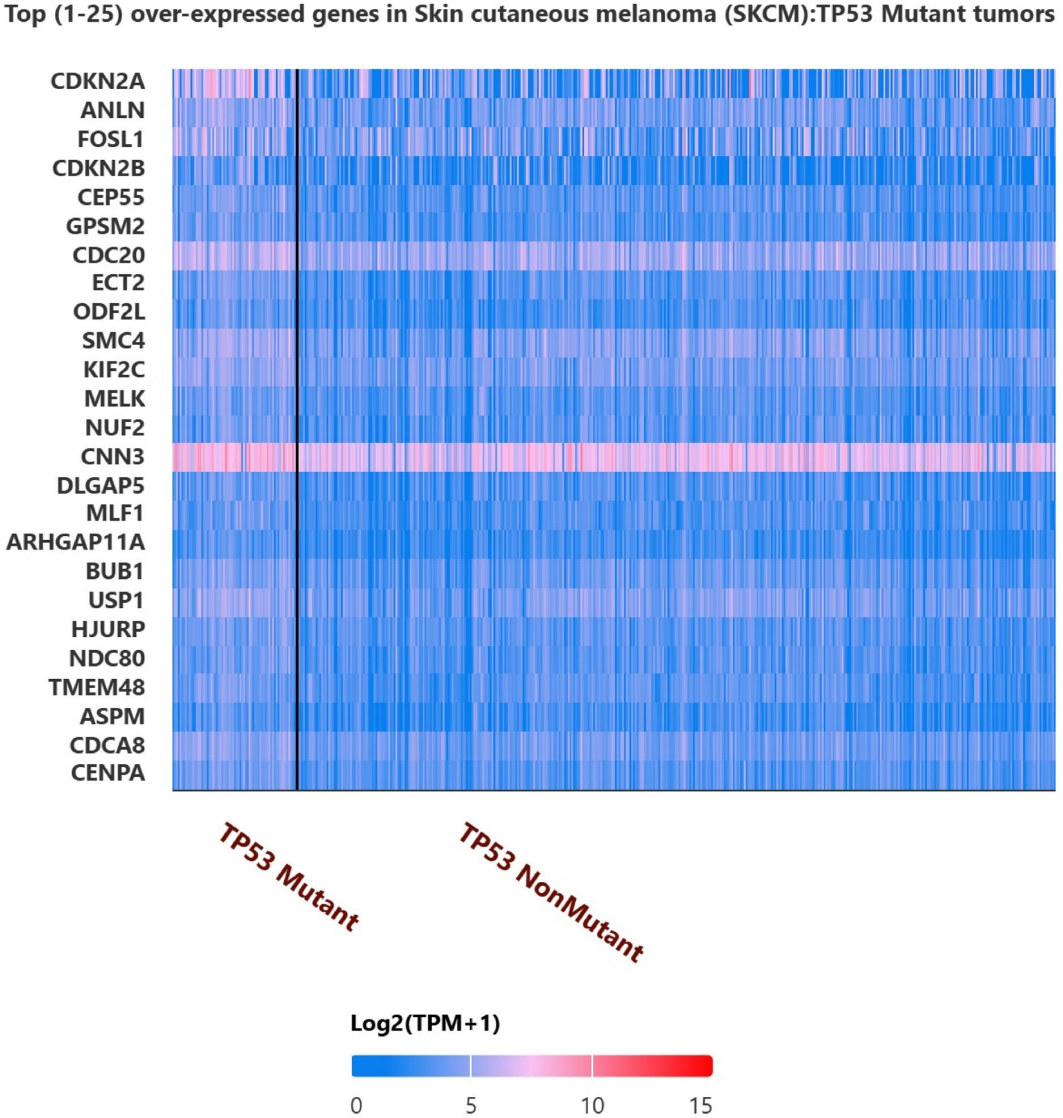
Differentially expressed genes in TP53-Mutant SKCM tumors.

Heatmap analysis revealed distinct expression patterns of MMPs and cell cycle-related genes in TP53-mutant SKCM tumors, pointing toward transcriptional reprogramming associated with genetic alterations (Fig. 7).

### Expression dynamics across tumor stages

**Fig. 8:**
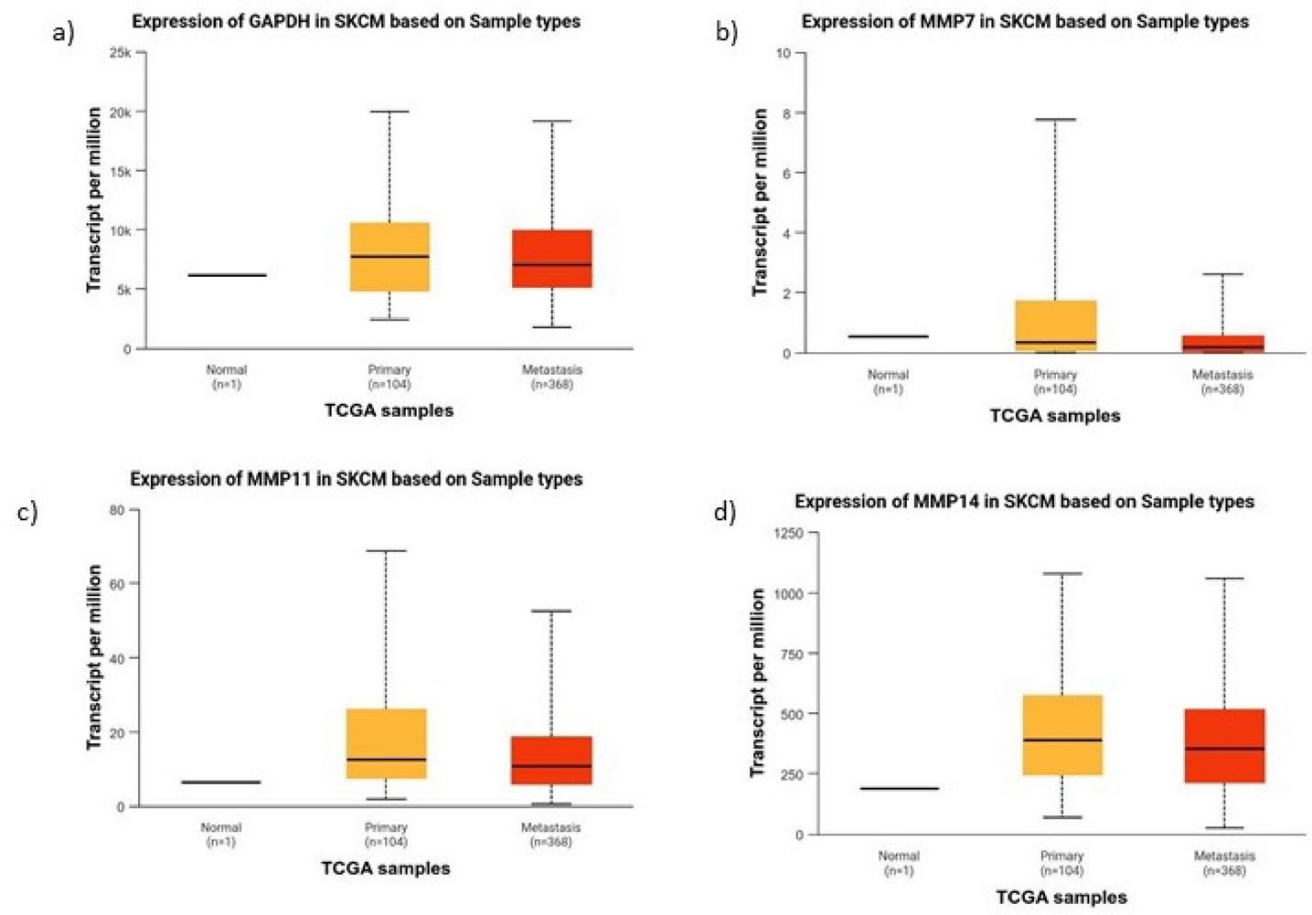
Differential expression of GAPDH, MMP7, MMP11, and MMP14 in SKCM based on TCGA sample types.

Differential expression analysis showed that MMP7 and MMP11 were elevated in primary tumors but downregulated in metastasis, whereas MMP14 remained consistently high, suggesting stage-specific roles (Fig. 8).

### Protein-level validation of MMP expression

**Fig. 9:**
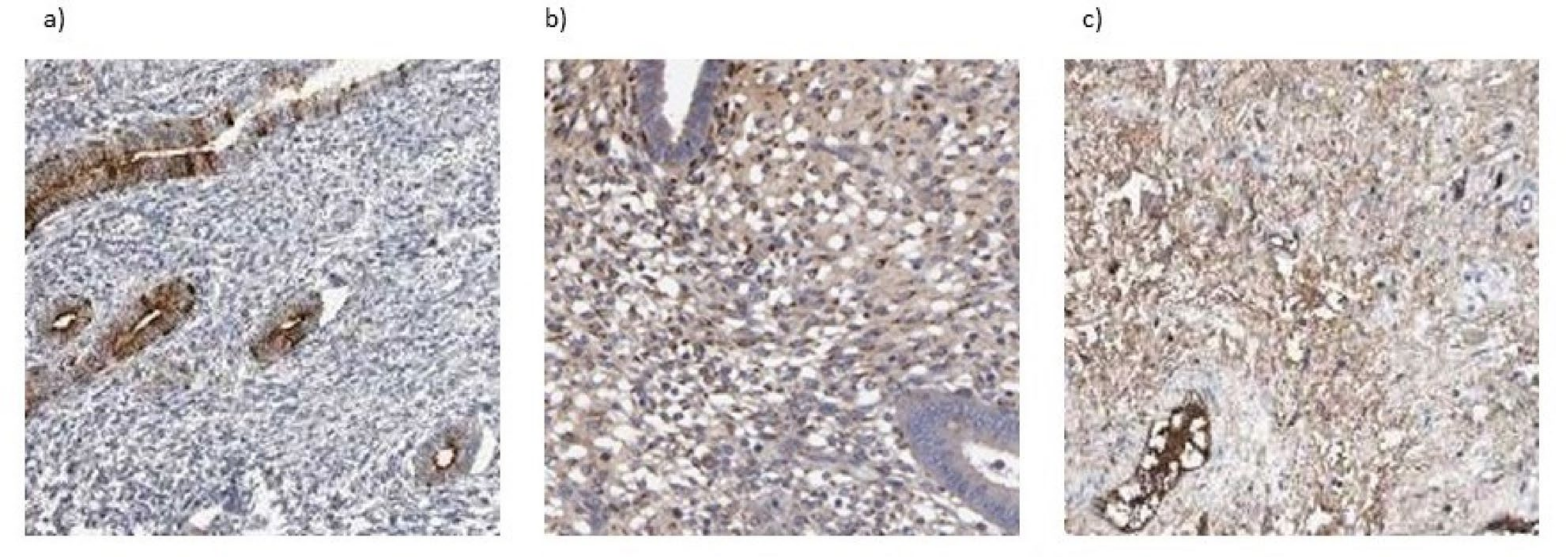
Immunohistochemical staining of MMP7, MMP11 and MMP14 in SKCM. (**a**) MMP7 (**b**) MMP11 (**c**) MMP14.

Immunohistochemistry from the Human Protein Atlas confirmed moderate to strong protein expression of MMP7, MMP11, and MMP14 in melanoma tissues, consistent with transcriptomic findings (Fig. 9).

### qRT-PCR validation in melanoma cell lines

qRT-PCR in A375 melanoma cells showed significant overexpression of MMP7, MMP11, and MMP14 compared to normal skin cells, further supporting their relevance in melanoma.

STRING-based PPI analysis revealed that MMP7, MMP11, and MMP14 were key hub proteins in ECM-related interaction networks. MMP7 forms dense cluster with multiple MMPs and ECM regulators, reinforcing its role in early ECM degradation. MMP11 connects not only with MMPs but also with collagens and processing enzymes, indicating its involvement in ECM reconstruction and activation pathways. In contrast, MMP14 serves as core mediator bridging ECM degradation and signaling, interacting extensively with TIMP1/2, CD44, and FURIN. These networks suggest that each MMP contributes uniquely to melanoma progression via distinct molecular mechanisms (Fig. 2).

We evaluated the prognostic significance of MMP7, MMP11, and MMP14 in SKCM using Kaplan–Meier survival analysis via GEPIA2 based on TCGA-SKCM patient data. Patients were divided into high-expression and low-expression groups using the median expression as the cutoff. The survival curves revealed that higher expression of MMP7, MMP11, and MMP14 was significantly associated with poor OS (Fig. 3):

- MMP7: High expression was correlated with reduced survival probability (HR = 1.1, Log-rank p = 0.012).
- MMP11: Elevated MMP11 expression showed strong negative correlation with OS (HR = 1.3, Log-rank p = 1.1e-14), indicating highly significant prognostic role.
- MMP14: Patients with high MMP14 expression had the most significant reduction in survival outcomes (HR = 1.6, Log-rank p = 0), suggesting its potential as a powerful prognostic biomarker.

Interestingly, elevated GAPDH expression was also linked to reduced survival, suggesting possible roles beyond its traditional use as a housekeeping gene (HR = 1.7, Log-rank p = 0). This suggested that GAPDH, beyond its role as a housekeeping gene, may have additional functional implications in SKCM progression.

Isoform structure analysis using GEPIA2 revealed functional diversity in MMP7, MMP11 and MMP14, highlighting their roles in ECM degradation and tumor progression.

- MMP7-001 contained PG_binding_1 and Peptidase_M10 domains, crucial for ECM remodeling and tumor invasion.
- MMP11-001 exhibited Peptidase_M10 and Hemopexin domains, supporting its role in proteolytic activity and substrate binding.
- MMP14 isoforms displayed multiple domains, including DUF3377, Hemopexin and Peptidase_M10, indicating a complex regulatory function in SKCM progression (Fig. 4).

ROC curve analysis was conducted to assess the diagnostic accuracy of MMP7, MMP11, and MMP14 in distinguishing SKCM from normal skin tissues. The AUC values obtained from our ROC plots were 0.945 for MMP7, 0.993 for MMP11, and 0.955 for MMP14. These AUC values demonstrate strong classification performance. We also clarify that the ROC analysis was performed using the same dataset (TCGA-SKCM) used for gene expression validation, without independent cross-validation or external dataset validation. Future studies should validate these findings in independent cohorts with appropriate cross-validation (Figure 5).

To explore potential co-expression relationships, Pearson correlation analysis was performed using GEPIA2 between GAPDH (control gene) and selected MMPs (MMP7, MMP11, MMP14) in TCGA-SKCM tumor samples. The results showed weak and non-significant correlations between GAPDH and MMP7 (R = 0.1, *p* = 0.12), GAPDH and MMP11 (R = 0.042, *p* = 0.52), and GAPDH and MMP14 (R = -0.04, *p* = 0.54). These findings suggest that GAPDH expression does not directly regulate or correlate with MMP expression in SKCM (Fig. 6).

Heatmap analysis of TP53-mutant vs. non-mutant SKCM tumors, conducted using UALCAN, revealed a distinct gene expression profile associated with TP53 mutations. Among the top 25 overexpressed genes, key oncogenic regulators such as CDKN2A, ANLN, FOSL1, CNN3, and DLGAP5 exhibited significantly higher expression in TP53-mutant samples. These genes are involved in cell cycle regulation, mitotic progression, and tumor proliferation, indicating that TP53 mutations may contribute to SKCMaggressiveness through dysregulated cell cycle pathways. The increased expression of CDKN2A suggests potential compensatory mechanisms for disrupted p53-mediated tumor suppression, while genes like DLGAP5 and ANLN highlight the role of mitotic instability in SKCM progression. These findings underscore the potential prognostic and therapeutic relevance of TP53-driven transcriptional changes in SKCM (Fig. 7).

Expression analysis of MMP7, MMP11 and MMP14 in TCGA-SKCM samples revealed distinct patterns across normal, primary and metastatic tissues. MMP7 and MMP11 were significantly upregulated in primary SKCM but showed reduced expression in metastatic samples, suggesting their involvement in early tumor invasion and progression. In contrast, MMP14 remained consistently elevated across primary and metastatic stages, indicating its persistent role in tumor progression and metastasis. These results suggest that MMP7 and MMP11 may serve as biomarkers for early-stage SKCM, while MMP14 could be a potential therapeutic target for both early and advanced melanoma (Fig. 8).

The IHC analysis from Human Protein Atlas confirmed protein-level expression of MMP7, MMP11 and MMP14 in SKCM tissues. MMP7 and MMP11 showed moderate cytoplasmic staining, while MMP14 exhibited stronger and more diffuse staining, suggesting its prominent role in tumor invasion and extracellular matrix remodeling. These findings align with transcriptomic (GEPIA/TCGA) and Western blot data, reinforcing the involvement of MMPs in SKCM progression and metastasis (Fig. 9).

To validate transcriptomic findings at the mRNA level, RT-qPCR was performed for MMP7, MMP11, and MMP14 in A375 melanoma cells versus normal skin cells. Consistent with the in silico analysis, all three MMPs exhibited significantly higher expression in melanoma cells. MMP14 showed the highest fold increase (~ 5.6-fold), followed by MMP11 (~ 4.2-fold) and MMP7 (~ 3.8-fold), normalized to GAPDH. These results confirm the overexpression of these MMPs at the transcript level and further support their involvement in SKCM progression.

## Discussion

Matrix metalloproteinases, particularly MMP7, MMP11, and MMP14, play pivotal roles in SKCM and SCC progression through their involvement in ECM remodeling. The dysregulation of MMPs facilitates tumor cell invasion, angiogenesis and immune evasion. Our findings demonstrated that high expression of these MMPs correlated significantly with poor overall survival in SKCM, reinforcing their prognostic relevance. A substantial number of differentially expressed genes between SCC and normal skin tissues, with MMP1, MMP10, MMP13, and MMP9 significantly upregulated, were identified. These MMPs are known for their pivotal role in ECM degradation, facilitating tumor invasion, metastasis, and stromal remodeling. The high logFC values of MMP1 and MMP10 underscore their dominant role in SCC pathogenesis. Additionally, MMP13 was strongly upregulated, reinforcing its function in ECM turnover and cancer invasiveness. A potential regulatory link between MMP9 and SLC12A5-AS1 highlights the relevance of non-coding RNAs in modulating MMP-mediated tumor progression. These findings align with previous literature reporting MMPs as critical mediators of cancer cell migration and invasion by dismantling ECM integrity^24,27,28^.

Conversely, widespread downregulation of keratin genes (e.g., KRTAP1-5, KRT26, KRT40) was observed, suggesting compromised epithelial barrier in SCC. Keratins are essential structural proteins that maintain epithelial homeostasis and resist mechanical stress. The suppression of these genes, including KRT15, KRT33A, and KRT31, reflects loss of skin integrity and differentiation, which likely contributes to increased fragility and enhanced tumorigenic potential. This is consistent with previous studies showing keratin gene downregulation in melanoma, further validating the common mechanisms of epithelial transformation and skin barrier disruption^29–31^.

Functional enrichment analysis revealed that dysregulated genes in SCC were significantly enriched in ECM-receptor interaction, focal adhesion, and proteoglycans in cancer pathways—all indicative of an altered ECM landscape. Genes such as LAMB3, LAMA3, and SPP1 were notably upregulated, pointing to aberrant ECM signaling and tissue architecture in SCC. These ECM-related genes promote cell motility, angiogenesis, and invasion, contributing to cancer progression. LAMB3, in particular, has been implicated in melanoma metastasis, aligning with our cross-cancer comparative findings^32,33^. We also observed enrichment in the IL-17 signaling pathway, a hallmark of chronic inflammation in the tumor microenvironment. Upregulated pro-inflammatory chemokines CXCL6 and CXCL5, along with ECM-degrading enzymes MMP1 and MMP13, create a feedback loop that sustains inflammation and facilitates tumor-supportive immune infiltration^34^.

A deeper look into SKCM datasets via TCGA validation revealed that MMP7, MMP11, and MMP14 also exhibited high expression in primary melanoma tumors. Notably, MMP14 remained consistently expressed in both primary and metastatic tumors, while MMP7 and MMP11 were predominantly expressed in the earlier stages. These findings were supported by UALCAN analysis, showing reduced expression of MMP7 and MMP11 in metastatic melanoma, whereas MMP14 remained elevated, implying a persistent role in tumor invasion and migration^35,36^. Survival analysis (via GEPIA2) demonstrated that high expression of MMP14 and MMP11 is associated with poorer overall survival in SKCM patients, suggesting their potential as prognostic biomarkers^37,38^. Interestingly, MMP14 showed the strongest association with poor outcomes, emphasizing its aggressive tumor-promoting behavior.

ROC analysis confirmed high diagnostic accuracy of MMP7, MMP11, and MMP14. These results strongly support their utility as diagnostic biomarkers for melanoma^39^. These findings are consistent with recent analyses demonstrating that overexpression of MMPs is not only associated with tumor aggressiveness but also predictive of immunotherapy responsiveness, particularly in cutaneous malignancies^40^. To evaluate diagnostic potential, ROC curve analysis revealed that MMP7 and MMP14 exhibit considerable AUC values, suggesting strong discriminative power between tumor and normal tissues. These findings are consistent with prior literature on MMP involvement in aggressive tumor behavior and align with recent work emphasizing the role of immune microenvironment remodeling in shaping therapy response. For instance, Zhang et al. highlighted how MMP-induced remodeling promotes immune checkpoint inhibitor resistance by hindering immune cell infiltration and facilitating immune evasion in skin cancers^41^.

Further, PPI analysis using STRING revealed robust interaction networks for MMP7, MMP11, and MMP14. MMP7 formed a dense cluster with other MMPs and inhibitors (e.g., TIMP1/2, SPP1, CD44), indicating its role in ECM degradation. MMP11 was linked to collagen family proteins (COL1A1, COL11A1) and ECM regulators like FURIN, suggesting involvement in collagen remodeling^42,43^. MMP14’s network displayed widespread interactions with ECM-remodeling enzymes and adhesion molecules, further confirming its central role in matrix regulation and metastasis. Additionally, IHC images from the Human Protein Atlas confirmed moderate to strong protein-level expression of MMP7, MMP11, and MMP14 in SKCM tissues, supporting transcriptomic findings. Western blot validation reinforced these observations at the protein level in tumor cell lines compared to controls.

Our study also revealed that MMP11 expression is associated with poorer prognosis in SKCM patients. This aligns with recent pan-cancer studies, such as that by Ye et al., who developed a plasma cell-based immune signature predictive of immunotherapy response. Their findings emphasize that tumors with enriched immune infiltration often shaped by ECM characteristics demonstrate heightened immunogenicity and better therapy outcomes^44^. This suggests that MMP expression could serve as a surrogate marker for tumor immune status and potentially guide patient stratification for immunotherapy.

This study provided an integrative transcriptomic and functional analysis of gene expression alterations in SCC and SKCM, identifying MMPs as central to both ECM remodeling and cancer progression. Alongside significant upregulation of MMP1, MMP10, MMP13, and MMP14 in SCC, we observed concurrent downregulation of keratin genes, reflecting loss of epithelial integrity and enhanced invasiveness. Beyond their structural role in ECM degradation, our findings align with emerging literature indicating that MMPs actively regulate TME. For instance, MMP14 interacts with immune-related components such as CD44 and FURIN, suggesting a role in modulating immune surveillance and cell adhesion. This is supported by recent multiomics studies demonstrating that MMP expression correlates with TME composition, immune cell infiltration, and immune checkpoint gene expression^14^. In addition to ECM degradation, MMPs may act as signaling mediators influencing angiogenesis and immune modulation. Notably, Zhang et al. demonstrated that specific MMPs, particularly MMP14, contribute to immune exclusion and resistance to anti-PD-1 therapy. Pharmacological inhibition of MMP14 restored immune cell infiltration and sensitized tumors to checkpoint blockade^41^. These findings further validate our results and support the exploration of MMP-targeted therapies in combination with immunotherapeutic agents.

MMP7 and MMP11, primarily overexpressed in early-stage melanoma, may influence the immunogenic landscape by regulating cytokine processing and enabling immune evasion. Meanwhile, MMP14’s consistent elevation in both primary and metastatic stages indicates a sustained role in shaping an immunosuppressive and pro-invasive TME. These findings are consistent with studies highlighting TME-driven resistance to immunotherapy, in which MMPs mediate ECM stiffening, immune exclusion, and CAF activation^14–18^.

Collectively, these observations reinforce the hypothesis that MMPs are not merely ECM degraders but are also functional regulators of the tumor–immune–stroma axis. Their dual roles highlight their utility as both biomarkers and therapeutic targets. Future research should explore the interplay between specific MMP isoforms and TME components across treatment stages to facilitate precision oncology in melanoma and SCC.

In addition to ECM degradation, MMPs play broader roles in tumor progression, including immune evasion, angiogenesis, and metastasis. MMP7 can inhibit immune responses by cleaving Fas ligand, while MMP11 suppresses chemokine-mediated immune cell recruitment. MMP14 contributes to both invasion and immune exclusion through ECM remodeling and CD44/FURIN interactions. Despite the failure of early broad-spectrum MMP inhibitors in clinical trials due to toxicity and lack of specificity, emerging strategies focus on isoform-specific targeting and combination therapies. Notably, combining selective MMP inhibitors with immunotherapy holds promise for enhancing immune cell infiltration and overcoming ECM-mediated resistance. Our findings suggest that MMP7 and MMP11 may serve as early-stage targets, whereas MMP14 could inform treatment strategies in advanced melanoma and SCC. Furthermore, recent studies highlight the potential of MMP-targeted therapies in synergy with immune checkpoint blockade, suggesting that MMP inhibition can remodel the tumor microenvironment to enhance immunogenicity^40,44^.

Although several ECM-related genes such as LAMB3, LAMA3, and SPP1 were significantly upregulated, we chose to focus on MMP7, MMP11, and MMP14 given their known roles in matrix degradation, immune evasion, and metastatic progression, which make them more clinically actionable as both biomarkers and therapeutic targets. Their positioning as central nodes in the protein–protein interaction network further supported their selection for downstream analysis.

### Limitations

One limitation of this study is that ROC curve analysis was performed on the same dataset used for gene discovery, without independent cross-validation or external validation, which may overestimate the diagnostic performance of MMP7, MMP11, and MMP14. Another limitation is the use of GAPDH both as a normalization control in qRT-PCR and as a prognostic marker in survival analysis. While its expression was stable across experimental cell lines, this dual role may introduce interpretive bias and should be validated using additional housekeeping genes in future studies.

## Conclusion

Our study highlights MMP7, MMP11, and MMP14 as key contributors to ECM remodeling in SCC and SKCM. These MMPs demonstrate potential as diagnostic and prognostic biomarkers and may serve as therapeutic targets, especially in the context of resistance to immunotherapy. Continued investigation of ECM-focused interventions could enhance treatment outcomes for skin cancer patients.

## Data Availability

The datasets generated and/or analyzed during the current study are available in the Gene Expression Omnibus (GEO) repository, under accession number GSE191334. Additional validation analyses were performed using public datasets from The Cancer Genome Atlas (TCGA) accessible via the UCSC Xena platform (https://xenabrowser.net/).

## Abbreviations

TCGA: Cancer genome atlas
GEO: Gene expression omnibus
TME: Tumor microenvironment
FSP1: Fibroblast-specific protein 1
SKCM: Skin cutaneous melanoma
SCC: Squamous cell carcinoma
